# Plant Kinesin-12: Localization Heterogeneity and Functional Implications

**DOI:** 10.3390/ijms20174213

**Published:** 2019-08-28

**Authors:** Sabine Müller, Pantelis Livanos

**Affiliations:** Center for Plant Molecular Biology, Auf der Morgenstelle 32, 72076 Tübingen, Germany

**Keywords:** cytokinesis, division plane determination, microtubules, gametogenesis, phragmoplast midzone

## Abstract

Kinesin-12 family members are characterized by an N-terminal motor domain and the extensive presence of coiled-coil domains. Animal orthologs display microtubule plus-end directed motility, bundling of parallel and antiparallel microtubules, plus-end stabilization, and they play a crucial role in spindle assembly. In plants, kinesin-12 members mediate a number of developmental processes including male gametophyte, embryo, seedling, and seed development. At the cellular level, they participate in critical events during cell division. Several kinesin-12 members localize to the phragmoplast midzone, interact with isoforms of the conserved microtubule cross-linker MICROTUBULE-ASSOCIATED PROTEIN 65 (MAP65) family, and are required for phragmoplast stability and expansion, as well as for proper cell plate development. Throughout cell division, a subset of kinesin-12 reside, in addition or exclusively, at the cortical division zone and mediate the accurate guidance of the phragmoplast. This review aims to summarize the current knowledge on kinesin-12 in plants and shed some light onto the heterogeneous localization and domain architecture, which potentially conceals functional diversification.

## 1. Introduction

The kinesin superfamily consists of heterogeneous microtubule-dependent motors that exist in all eukaryotes [1]. Kinesins have been significantly expanded in higher plants. For instance, in *Arabidopsis* 61 members have been found. The large number of kinesins implies that they participate in a variety of processes including plant specific ones [2,3]. Kinesins are divided into 14 families; however, there are some “orphans” that are not classified in any of these [3,4]. The phylogeny of kinesins is mostly based on their motor domain sequence which may be located at the amino (N)-terminus, the central region of the molecule, or at the carboxy (C)-terminus [1,3]. The typical architecture of kinesins also includes the neck, the stalk, which is a region responsible for dimerization and interactions containing coiled-coil domains, and the tail, which binds to specific cargo [3,5]. The directionality of kinesins along microtubules differs between families and is usually related to the position of the motor domain [3].

**Figure 1:**
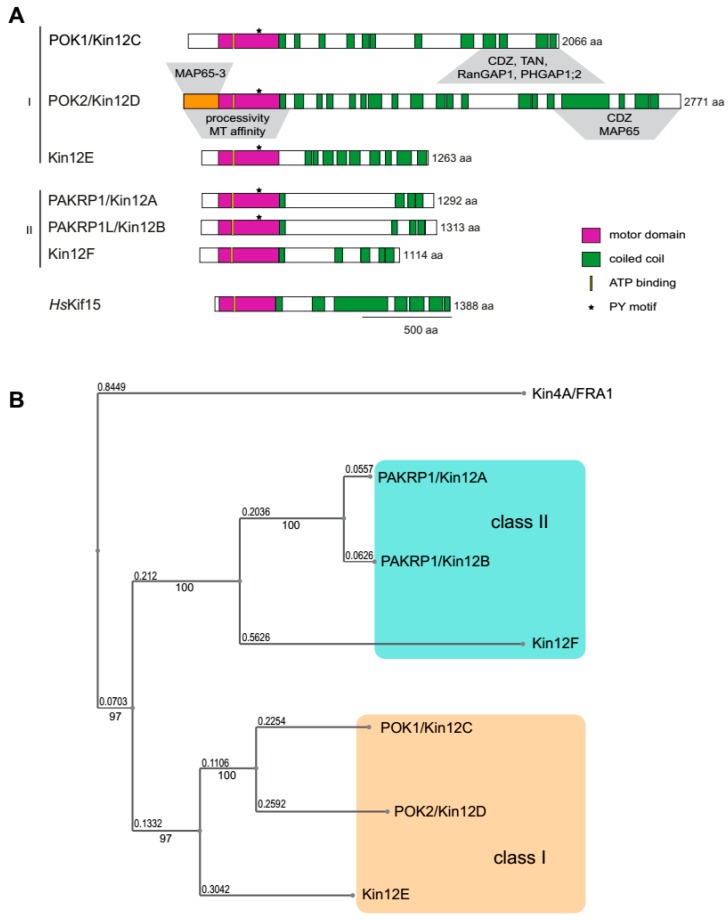
Protein domain architecture and phylogeny of *Arabidopsis* kinesin-12. (**A**) *Arabidopsis* kinesin-12 (Kin12A–F) are categorized in two classes: Class I includes the large PHRAGMOPLAST ORIENTING KINESINS 1 (POK1)/Kin12C and POK2/Kin12D as well as the yet uncharacterized Kin12E; class II consists of PAKRP1/Kin12A, PAKRP1L/Kin12B, and Kin12F. Kinesin-12 members have an N-terminal motor domain indicative of microtubule plus-end directed motility. Unlike the human kinesin-12 HsKif15 and kinesin12F, all other plant kinesin-12 members possess a proline-tyrosine (PY) motif (asterisk), potentially involved in the regulation of motor activity and protein stability. Regions that appear to be important for targeting or interaction with other proteins are depicted above (grey trapezoid). Note the difference in size between the N-terminal region upstream of the motor domain in the plant orthologs and HsKif15. This disordered region is thought to facilitate interactions with other proteins. In the case of POK2/Kin12D, it has been shown that this region (orange bar) serves as a specific binding site to MICROTUBULE-ASSOCIATED PROTEIN 65-3 (MAP65-3) which mediates POK2 recruitment to microtubules. (**B**) Phylogenetic tree of the *Arabidopsis* Kinesin-12 family. Kinesin-4/FRA1 was used as an outgroup. Amino acid sequences of full-length proteins were aligned using the multiple sequence alignment program MAFFT and the tree was constructed using the neighbor-joining method and a 1000 bootstrap resampling value, and visualized using Archeopteryx [18,19]. Bootstrap values and branch lengths are displayed.

Kinesins that belong to family 12 share some common features with the animal kinesin family member 12 (Kif12) which was the founding member [5]. In general, kinesin-12 members are characterized by the presence of an N-terminal motor domain, a neck β-sheet, and by the homology of the amino acid sequence in their C-terminal, which is abundant in coiled-coil domains (Figure 1) [5]. Plant kinesin-12 PHRAGMOPLAST ORIENTING KINESINS (POKs) are among the largest kinesins that have been reported so far [6,7,8]. Unlike their animal orthologs, upstream of the motor domain, plant kinesin-12 members possess an extraordinarily long N-terminal disordered region (Figure 1), which facilitates binding to various partners with high specificity [9]. Kinesins of this family have in common a microtubule plus end-directed motility [5,8,10,11,12]. In animals, the kinesin-12 members, such as Kif15 and Kif12, are associated with mitosis, mediating and/or promoting the assembly of the bipolar spindle, and chromosome movement, and they have been also implicated in cytokinesis [13,14,15]. In addition to the critical role in cell proliferation, animal kinesin-12 members are expressed in post-mitotic neurons mediating axon growth and neuronal migration [16,17]. 

**Figure 2:**
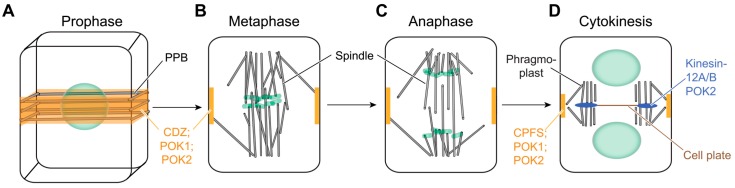
Depiction of localization patterns of characterized *Arabidopsis* kinesin-12 throughout mitosis and cytokinesis. (**A**) POK1/Kin12C and POK2/Kin12D (orange) colocalize with microtubules (grey) of the preprophase band (PPB) in prophase. (**B**,**C**) Following PPB disassembly at the end of prophase, POKs are tethered to the plasma membrane occupying a region at the cell cortex that delineates the future division plane (cortical division zone, CDZ; orange) throughout the metaphase and anaphase. (**D**) During cytokinesis, PAKRP1/Kin12A, PAKRP1L/Kin12B, and POK2/Kin12D decorate the phragmoplast midzone (blue), contributing to the stabilizing of the interdigitated microtubules there. At the end of cytokinesis, phragmoplast peripheral microtubule plus-ends extend towards the lateral walls. POK1/Kin12C and POK2/Kin12D residing there mediate the guidance of the phragmoplast and the accurate cell plate (brown) navigation to the preselected cell plate fusion site (CPFS, orange). Image (**A**) shows a 3D-representation of the cell, while (**B**–**D**) depict median cell planes.

In plants, the kinesin-12 members that have been characterized so far are implicated mainly in cell division. Contrary to the majority of their family animal orthologs, a number of plant kinesins decorate the midzone of the cytokinetic apparatus and/or the cortical division zone, defining the future division plane (Figure 2). Over the years, experiments have provided evidence showing that these kinesin-12 members interact with a variety of microtubule-associated proteins as well as with others that interfere with their spatiotemporal pattern and function [20,21]. Although our understanding is mostly based on data derived from higher plants, recent studies dealing with the characterization of members of the kinesin superfamily in other species point towards similar, but not identical cell division-related functions to their higher plant orthologs [3]. This emerging diversification, combined with recent data reporting novel functions of kinesin-12 orthologs, supports the view that this understudied family is much more heterogeneous than initially thought. In the present review, we summarize previous and current findings concerning kinesin-12 and highlight their significance for growth and development in plants. 

## 2. Plant Kinesin-12: An Overview

### 2.1. Redundancy, Conserved and Novel Functions 

In *Arabidopsis*, there are six members of kinesin-12, in rice seven have been identified, and in *Populus* five [22,23]. In addition, the fern *Marsilea* possesses six predicted kinesin-12 members [24], but the family is greatly amplified in *Physcomitrella* which contains 20 genes encoding for kinesin-12, indicating extensive redundancies of these motor proteins in *Physcomitrella* [3,25]. Phylogenetic analysis shows that in *Arabidopsis* and *Physcomitrella,* the kinesin-12 family is comprised of two classes (I and II), as in both species kinesin-12 fall into two distinct clusters (Figure 1B) [3,8,12]. Nevertheless, most of our knowledge concerning kinesin-12 originates from studying *Arabidopsis*. PHRAGMOPLAST-ASSOCIATED KINESIN-RELATED PROTEIN 1 (PAKRP1/Kin12A) and PAKRP1-LIKE PROTEIN (PAKRP1L/Kin12B) were the first characterized plant kinesin-12 members, and therefore, they were named Kinesin 12A and Kinesin 12B, respectively, according to the kinesin nomenclature [4]. PAKRP1/Kin12A and PAKRP1L/Kin12B belong to class II and regulate the organization of the phragmoplast during male sporogenesis [26,27,28]. The class I proteins POK1/Kin12C and POK2/Kin12D mediate accurate cell plate positioning (Figure 1) [6,7,8,12]. Although Kin12E (encoded by *At3g44050*, class I, Figure 1) and Kin12F (encoded by *At3g20150*, class II, Figure 1) have not yet been characterized, transcript profiling revealed that both are upregulated during mitosis, suggesting a cell division-related role [29]. More recently, kinesin-12, *Nicotiana tabacum* KINESIN RELATED PROTEIN (NtKRP) that belongs to the subclade of *Arabidopsis* PAKRP1/Kin12A and PAKRP1L/Kin12B, was identified. This kinesin has microtubule binding ability, but also binds CYCLIN DEPENDENT KINASE A and seems to be necessary for G2/M progression [30]. It acts towards seed and embryo size regulation, as knocking down of *NtKRP* results in smaller embryos and seeds due to fewer cells [30]. Furthermore, it functions in concert with NtRLP17 (*Nicotiana tabacum* RIBOSOMAL-LIKE PROTEIN 17), revealing a previously unknown link between kinesins and ribosomal proteins [31]. Therefore, kinesin-12 family members are apparently closely associated with cell division. Indeed, most family representatives in higher plants are present in meristems. For instance, POK1/Kin12C and POK2/Kin12D display similar distribution patterns and are expressed in root meristems, leaf primordial, and young leaves, among other tissues. Since defects in cell divisions directly affect cell morphology and plant growth, the abolishment of kinesin-12 functions do introduce severe defects. In *pok1pok2* double mutants, impairment of proper cell wall positioning is detected early in embryogenesis leading to misshapen embryos, and adult plants display irregular cell shapes, are dwarfed, and although still fertile, produce a vastly reduced number of seeds [8]. Double mutants devoid of PAKRP1/Kin12A and PAKRP1L/Kin12B produce very few seeds due to male gametophytic defects, whereas no detectable aberrations are present in developing embryos and plants at the vegetative stage [28]. 

### 2.2. Kinesin-12 and Cytokinesis

**Figure 3:**
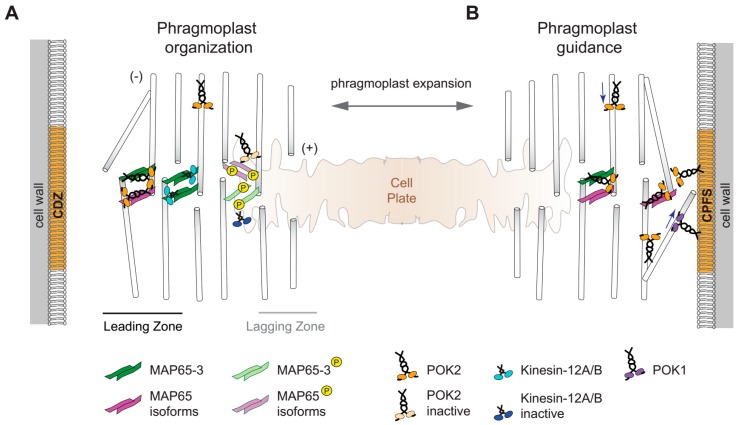
*Arabidopsis* kinesin-12 members mediate phragmoplast stability, expansion, and its accurate guidance towards the preselected cell plate fusion site during cytokinesis. (**A**) The double-sided arrow shows the direction of the phragmoplast expansion. The expansion of the phragmoplast is critical for the guidance of cytokinetic vesicles to fuse within the division plane leading to the formation of the cell plate. (**A**) The phragmoplast consists of two antiparallel subsystems of microtubules with some plus ends (+) overlapping at the midzone. The phragmoplast expands toward the cell cortex with continuous polymerization of microtubules at the leading zone and simultaneous depolymerization of microtubules at the lagging zone. Interactions of microtubule plus-end directed PAKRP1/Kin12A, PAKRP1L/Kin12B, and POK2/Kin12D with MAP65-3 or other MAP65 isoforms mediate their recruitment to the phragmoplast midzone, which in turn aids in phragmoplast stability and balanced expansion. Phosphorylation triggers detachment of MAP65 isoforms from microtubules regulating their turnover. The exact mode of kinesin-12 activity/regulation at the midzone is currently unknown during phragmoplast microtubule organization; however, midzone kinesin-12 homologs, assumed to be no longer associated with MAP65 isoforms (“inactive kinesin-12”), are depicted in a different color. (**B**) At later stages of cytokinesis, peripheral microtubules extent towards the cell cortex. POK1/Kin12C and POK2/Kin12D residing at the cortical division zone (CDZ) likely interact with these microtubules and walk towards their plus ends (blue arrow). This leads to the guidance of the phragmoplast and the anchoring of the cell plate to a median region of the cortical division zone, the cell plate fusion site (CPFS).

Unlike functions of their animal orthologs, to date, kinesin-12 functions at the spindle of higher plants are not documented, although the presence of several *Physcomitrella* kinesins-12 members at the metaphase spindle has been reported [3]. However, the spatiotemporal patterns of some higher plant representatives show a strong association of kinesin-12 orthologs with the cytokinetic apparatus i.e., the phragmoplast (Figure 2 and Figure 3). The bipolar phragmoplast consists of microtubules and actin filaments. The main function of the phragmoplast is to guide fusion-competent cytokinetic vesicles towards its midzone in which the plus ends of a fraction of antiparallel microtubules overlap (Figure 2 and Figure 3). During phragmoplast expansion towards the cell cortex, microtubules polymerize at its leading edge and depolymerize from the central lagging zone upon vesicle fusion and cell plate biosynthesis (Figure 3) [32]. *Arabidopsis* PAKRP1/Kin12A and PAKRP1L/Kin12B as well as POK2/Kin12D emerge during late anaphase and label the midzone of the phragmoplast until its disassembly at the end of cytokinesis (Figure 2 and Figure 3) [7,27]. Immunolocalization experiments also show OsKINESIN12A at the phragmoplast midzone in rice [22], supporting the notion that the association with the midzone might be a common feature of kinesin-12; although this requires further investigation. 

Interestingly, those *Arabidopsis* kinesin-12 members that localize to the midzone of the phragmoplast also share the microtubule cross-linker MICROTUBULE-ASSOCIATED PROTEIN 65-3 (MAP65-3)/PLEIADE (PLE) as an interaction partner, aiding the maintenance of the bipolar phragmoplast. MAP65 family members play a role in microtubule stabilization (Figure 3) [33,34]. In particular, MAP65-3 forms cross-bridges between the interdigitating microtubules of the phragmoplast and this cross-linking function is essential for phragmoplast integrity and for cytokinesis [34,35]. In *ple-2* mutants, phragmoplasts display considerably wider midzones, subsequently leading to cytokinesis failure and the formation of incomplete cell walls or stubs and multinucleated cells [35]. Notably, MAP65-3/PLE emerges at the overlap of the antiparallel microtubules in the anaphase spindle before PAKRP1/Kin12A and PAKRP1L/Kin12B arrive at the phragmoplast midzone [36]. MAP65-3 seems to be essential for midzone retention of PAKRPs as midzone association of PAKRP1/Kin12A was abolished in *dyc283* MAP65-3 mutants [36]. PAKRP1/Kin12A and PAKRP1L/Kin12B are decorating the interdigitating plus ends of phragmoplast microtubules within the division plane (Figure 3) [27,28]. The *kin12akin12b* double mutants fail to organize a functional phragmoplast, as the microtubules between the daughter nuclei do not acquire a bipolar arrangement [28]. However, this is only observed in dividing microspores whereas somatic cells undergo unperturbed cytokinesis, suggesting functional redundancy in these cells. A recently characterized candidate that might counterbalance the loss of PAKRP1/Kin12A and PAKRP1L/Kin12B function in somatic cells of the respective double mutants is kinesin POK2/Kin12D. It begins to accumulate at the phragmoplast midzone during the transition to cytokinesis and continuously localizes there until the disappearance of the phragmoplast (Figure 2) [7]. In a motility depended manner, POK2/Kin12D reaches the midzone where it interacts with MAP65-3 and likely some of its isoforms (Figure 3) [7]. The interaction with MAP65-3 is probably confining POK2/Kin12D at the midzone as it is diminished in *ple-2* mutants [7]. MAP65-3 seems to have a specific affinity for the intrinsically disordered N-terminal region of POK2/Kin12D that directly precedes the motor domain (Figure 1). Moreover, POK2/Kin12D binds to various MAP65 family members including MAP65-3 via its C-terminal domain (Figure 1), which fine-tunes the midzone localization of POK2/Kin12D (Figure 3) [7]. Most likely, it is this capacity of the POK2/Kin12D C-terminal domain to bind various MAP65 isoforms that retains it at the midzone, even in the absence of MAP65-3/PLE. These various MAP65 isoforms exhibit distinct localization patterns during cytokinesis, decorating the entire phragmoplast or only regions of it. To date experiments investigating the genetic interactions of kinesin-12 with MAP65 members other than MAP65-3 are still needed to untangle functional redundancies. Thus, the topological redundancy of several kinesin-12 members at the midzone might essentially provide functional phragmoplast organization and expansion. Along these lines, it would be interesting to examine whether similar interaction modules exist between kinesin-12 and MAP65 in basal land plants. The kinesin-12 family in *Physcomitrella* is remarkably expanded, containing sixteen members that belong to class I (related to POKs) and four to class ΙΙ (related to PAKRP1/Kin12A and PAKRP1L/Kin12B), whereas at least three out of the five PpMAP65 members produce severe phenotypes upon simultaneous depletion (Figure 1) [3,12,37]. 

Lessons from animal kinesin-12 orthologs evidenced their microtubule bundling and sliding activity [10,13,14,38]. Although mutations of either single *PAKRP1/kinesin12A* or *PAKRP1L/kinesin12B* do not obviously interfere with the organization of the phragmoplast [28], the phragmoplast phenotype of *kin12akin12b* double mutants in microspores clearly demonstrate a role for the encoded proteins in stabilizing interdigitating microtubules within the phragmoplast midzone, whereas a potential role of POK2/Kin12D in the organization of the phragmoplast is currently masked by unresolved redundancies as *pok2* single mutants also exhibit wild-type looking phragmoplast arrays [7]. Although phragmoplast organization is intact in cytokinetic root cells of *pok2* single and *pok1pok2* double mutants, kymograph analysis revealed a significantly reduced expansion rate by the lack of POK2/Kin12D function, supporting a role of POK2/Kin12D in the timely phragmoplast expansion [7].

Remarkably, the slowdown of the phragmoplast expansion rate during cytokinesis does not result in detectable defects in cell plate formation ([6]; Stierhof and Müller, unpublished data). In contrast, it was shown, using the well-known cell-plate associated syntaxin/Qa-SNARE KNOLLE and callose staining that, in the absence of PAKRP1/Kin12A and PAKRP1L/Kin12B, disorganization of phragmoplasts affect the delivery of cytokinetic vesicles ultimately leading to the failure of cytokinesis in microspores [28]. However, regardless of the reported presence of specific cargo-associated tail domains in plant (e.g., kinesin-12 NtKRP) or in animal orthologs, there are no data supporting a direct role of PAKRP1/Kin12A and PAKRP1L/Kin12B or other plant kinesin-12 members in vesicle trafficking [15,30]. 

Without a doubt, intact phragmoplast organization is necessary for proper cell plate development [39,40]. The reorganization of phragmoplast microtubules is greatly controlled by the phosphorylation of MAP65 isoforms resulting in their inactivation and detachment from microtubules that subsequently depolymerize [41]. The phosphorylation is mediated by a well-characterized mitogen activated protein kinase (MAPK) signaling pathway. The recruitment and activation of the respective MAPK cascade requires tissue-specific isoforms of the kinesin-7 family HINKEL/NUCLEUS AND PHRAGMOPLAST LOCALIZED PROTEIN KINASE ACTIVATING KINESIN LIKE PROTEIN (NACK)/TETRASPORE [21]. Interestingly, the IMPORTIN-β IMB4 regulates the activity and stability of the kinesin-4 FRAGILE FIBER (FRA) 1 during development through interaction with a specific PY motif in its motor domain [42]. Among the kinesins tested in this study, the kinesin-7 NACK1 and the kinesin POK1/Kin12C contain the PY motif and their motors bind IMPORTIN-β, suggesting this might be a broad regulatory mechanism [42]. Yet, an effect on the activity or stability of POK1/Kin12C or NACK1 was not examined. Interestingly, the *Arabidopsis* kinesin-12 members, except Kin12F, contain the PY motif in their motor domains, although localization patterns are quite diverse (Figure 1). Notably, the POK1/Kin12C is not present at the phragmoplast midzone during cytokinesis, while POK2/Kin12D, PAKRP1/Kin12A and PAKRP1L/Kin12B are recruited there (Figure 2 and Figure 3). Therefore, whether IMPORTIN-β interferes with the activity and spatiotemporal patterns of kinesin-12 needs further investigation. 

One possible explanation regarding the regulation of kinesin-12 activity might be that the association with the phragmoplast midzone solely relies on MAP65 activity/inactivity cycles. Alternatively, kinesin-12 themselves might be direct substrates of the NACK-PQR MAPK signaling components. It is worthwhile mentioning that recently POK2/Kin12D was predicted as a putative target of AURORA kinase [43]. AURORAs are localizing to the phragmoplast [44,45] although the prediction was related to the cortical division zone targeting (see below) of POK2/Kin12D.

Interestingly, another signaling module involving PAKRP1/Kin12A, PAKRP1L/Kin12B, and TWO IN ONE (TIO) kinase is acting probably redundantly with the NACK-PQR pathway in male meiocytes [46]. TIO kinase and its interactors are thought to regulate the reorganization of phragmoplast microtubules [46]. Although these seem to be separate signaling pathways, they might converge on shared signaling components. TIO is functioning in sporophytic and gametophytic cell types and probably also has an essential role in somatic cytokinesis, since TIO RNAi seedlings exhibit multinucleated cells with incomplete cell walls [47,48].

### 2.3. Kinesin-12 POKs Are Core Components of the Cortical Division Zone

POK2/Kin12D not only supports the expansion of the phragmoplast midzone but also performs additional critical functions together with POK1/Kin12C (Figure 2 and Figure 3). Throughout cell division, both localize specifically at the cortical division zone. They arrive at the preprophase band in a microtubule dependent manner [6,7]. Following the disassembly of the microtubule preprophase band in late prophase, they remain tethered to the plasma membrane region that delineates the future division plane throughout mitosis and cytokinesis (Figure 2). There they act as scaffolds retaining other proteins residing at the cortical division zone [6,7,21]. POKs emerge at the preprophase band independently of TANGLED (TAN) and Ran GTPase-activating protein 1 (RanGAP1); however, the localization of the latter two at the cortical division zone is abolished in dividing cells of *pok1pok2* double mutants after preprophase band disassembly [20]. Interaction of POK1/Kin12C with TAN and RanGap1 is mediated by the C-terminal domain of POK1/Kin12C (Figure 1) [49,50,51]. Similarly to the POK1/Kin12C C-terminus, the POK2/Kin12D C-terminal domain is sufficient to identify the division site, indicating the existence of critical motifs that mediate targeting to this specific site of the cell cortex (Figure 1) [7]. It is likely that phosphoregulation might mediate anchoring of POK1/Kin12C and POK2/Kin12D at the plasma membrane, as well as the binding of TAN and RanGap1 to POK1C [6]. Regarding the mode of this regulation, only hypotheses can be made. TON2/FASS Protein Phosphatase 2A complex, which drives the assembly of the preprophase band, might be an obvious upstream regulatory candidate as it is present at the cortical division zone from preprophase until metaphase. Moreover, phosphatase inhibitors or genetic interference with TON2 function prevent cortical recruitment of TAN and RanGap1 entirely [49,50,51]. Interestingly, POK1/Kin12C still emerges, although delayed, and decorates the cortical division zone in the dividing cells of the preprophase band-less triple mutants *trm678*, further underpinning its role as a core component of the division plane, the setup of which is accelerated by the preprophase band [52].

Two more POK1/Kin12C interactors are the Rho GTPase of plants (ROP) GAPs PHGAP1 and PHGAP2 containing a pleckstrin-homology (PH) domain at their N-terminus [53]. In meristematic tissues, they exhibit preferential enrichment at the cortical division zone upon metaphase until the end of cytokinesis. The latter is abolished when POKs are missing but the interplay of PHGAPs with these kinesins probably involves feedback loops as YFP-POK1 cortical rings are frequently mislocalized in *phgap1phgap2* mutants, which also display moderate cell-wall mispositioning defects. Not surprisingly, this conspicuous accumulation of PHGAPs points to their interference with ROP activities, which are known to regulate the organization of the cytoskeleton and vesicle trafficking, and to PHGAPs plausible implication in the assembly or the maintenance of the actin depleted zone [20,53]. Notably, constructs driving the expression of POK1/Kin12C and POK2/Kin12D alone can rescue the double mutant phenotype, demonstrating that POK1/Kin12C and POK2/Kin12D have redundant functions at the cortical division zone, i.e., a scaffolding function and guidance of the phragmoplast [6,7].

### 2.4. An Update on Phragmoplast Guidance: The Primary Role of POKs

In dividing cells of *pok1pok2* double mutants, the assembly of the preprophase band takes place on time and the initiation of the phragmoplasts occurs between the daughter nuclei as expected. However, soon after, the phragmoplast deviates from the anticipated path as provided by the positional information of the preprophase band [6]. Consequently, the cell plate inserts at the parental cell wall in a random position, showing that the simultaneous lack of both POK1/Kin12C and POK2/Kin12D causes the failure in phragmoplast/cell plate guidance to the cortical division zone [6,8]. During cytokinesis, the phragmoplast microtubules polymerize at the margins of the phragmoplast with their plus ends extending towards the cortical division zone (Figure 3). POK1/Kin12C and POK2/Kin12D at the cortical division zone might bind to these peripheral microtubules to navigate the expanding phragmoplast at least in late cytokinesis (Figure 3). On the basis of their sequence, both are predicted to be plus-end directed, and that was recently confirmed for POK2/Kin12D motor [11,20]. Evidence favoring the above hypothesis of interaction between POKs and peripheral microtubules is that the C-terminal regions of both POK1/Kin12C and POK2/Kin12D mediate their attachment to the cortical division zone. However, expression of only the C-terminal fragments of POK1/Kin12C or POK2/Kin12D failed to complement the double mutant phenotype [6,7,21]. This speaks for a critical contribution of POK2/Kin12D and possibly the POK1/Kin12C functional motor domain at the cortical division zone [7]. In a recently proposed model, POKs’ binding to peripheral microtubules and moving towards their plus ends pushes against the expanding phragmoplast (Figure 3) [11,20]. The forces generated might be counterbalanced by minus-end directed kinesins [20,54]. Such a tug-of-war mode of phragmoplast guidance might fine-tune the approach of the phragmoplast and facilitate smooth anchoring of the cell plate within the preselected cell plate fusion site [11,20].

Notably, actomyosin is also widely implicated in the alignment of the cell plate to the preselected position. This has been shown in phrarmacological studies as well as in mutants devoid of MYOSIN XI in *Arabidopsis* [55,56,57,58]. Interestingly, animal kinesin-12 members interact with myosin isoforms [59]. For instance, *Dictyostelium* Kif12 is known to mediate translocation of MYOSIN II to the cortex of the dividing cells during metaphase [15]. However, to date, there is no clear evidence showing that POKs at the cortical division zone perform scaffolding or guidance-related functions via an interaction with the actomyosin cytoskeleton. In *Physcomitrella*, loss of MYOSIN VIII, which associates with microtubule plus ends and displays a localization pattern reminiscent of *Arabidopsis* POK2/Kin12D, leads to cell plate guidance defects [60]. On the basis of our current knowledge, a POK-related function has not been attributed to any of the 20 *Physcomitrella* kinesin-12 orthologs [3,24], but given that the great majority of them belong to the POK-related class I, this possibility must not be excluded [3,12].

## 3. Conclusions

The data reported so far point towards the important roles of class I kinesin-12 POKs in the timely establishment of the cortical division zone and the guidance of the phragmoplast to the cell plate fusion site in cytokinesis. Members of both POK-related class I and PAKRP-related class II regulate phragmoplast organization and/or tune phragmoplast stability. Accumulated evidence shows a variety of interactors but still our knowledge regarding the mode of kinesin-12 regulation and the existence of potential redundancies is limited. Further work is required to solve the redundancies of the *Arabidopsis* homologs as well as of the basal plant protein orthologs. This knowledge will improve our understanding on kinesin-12 spatiotemporal patterns, the diversification of their functions, and the extent of conserved interactions that kinesin-12 entertain, especially in light of the phylogenetic relationships between family members among and within species.

## References

[1] Cai G., Cresti M. (2012). Are kinesins required for organelle trafficking in plant cells?. Front. Plant Sci..

[2] Reddy A.S.N., Day I.S., Liu B. (2011). Microtubule Motor Proteins in the Eukaryotic Green Lineage: Functions and Regulation. The Plant Cytoskeleton, Advances in Plant Biology 2.

[3] Miki T., Naito H., Nishina M., Goshima G. (2014). Endogenous localizome identifies 43 mitotic kinesins in a plant cell. Proc. Natl. Acad. Sci. USA.

[4] Lawrence C.J., Dawe R.K., Christie K.R., Cleveland D.W., Dawson S.C., Endow S.A., Goldstein L.S.B., Goodson H.V., Hirokawa N., Howard J. (2004). A standardized kinesin nomenclature. J. Cell Biol..

[5] Miki H., Okada Y., Hirokawa N. (2005). Analysis of the kinesin superfamily: Insights into structure and function. Trends in Cell Biol..

[6] Lipka E., Gadeyne A., Stöckle D., Zimmermann S., De Jaeger G., Ehrhardt D.W., Kirik V., Van Damme D., Müller S. (2014). The phragmoplast-orienting kinesin-12 class proteins translate the positional information of the preprophase band to establish the cortical division zone in *Arabidopsis thaliana*. Plant Cell.

[7] Herrmann A., Livanos P., Lipka E., Gadeyne A., Hauser M.-T., Van Damme D., Müller S. (2018). Dual localized kinesin-12 POK2 plays multiple roles during cell division and interacts with MAP65-3. EMBO Rep..

[8] Müller S., Han S., Smith L.G. (2006). Two kinesins are involved in the spatial control of cytokinesis in *Arabidopsis thaliana*. Curr. Biol..

[9] Seeger M.A., Rice S.E. (2013). Intrinsic disorder in the kinesin superfamily. Biophys. Rev..

[10] Drechsler H., McHugh T., Singleton M.R., Carter N.J., McAinsh A.D. (2014). The Kinesin-12 Kif15 is a processive track-switching tetramer. eLife.

[11] Chugh M., Reißner M., Bugiel M., Lipka E., Herrmann A., Roy B., Müller S., Schäffer E. (2018). Phragmoplast orienting kinesin 2 is a weak motor switching between processive and diffusive modes. Biophys. J..

[12] Shen Z., Collatos A.R., Bibeau J.P., Furt F., Vidali L. (2012). Phylogenetic analysis of the kinesin superfamily from *Physcomitrella*. Front. Plant Sci..

[13] Sturgill E.G., Das D.K., Takizawa Y., Shin Y., Collier S.E., Ohi M.D., Hwang W., Lang M.J., Ohi R. (2014). Kinesin-12 Kif15 targets kinetochore fibers through an intrinsic two-step mechanism. Curr. Biol..

[14] Drechsler H., McAinsh A.D. (2016). Kinesin-12 motors cooperate to suppress microtubule catastrophes and drive the formation of parallel microtubule bundles. Proc. Natl. Acad. Sci. USA.

[15] Lakshmikanth G.S., Warrick H.M., Spudich J.A. (2004). A mitotic kinesin-like protein required for normal karyokinesis, myosin localization to the furrow, and cytokinesis in *Dictyostelium*. Proc. Natl. Acad. Sci. USA.

[16] Liu M., Nadar V.C., Kozielski F., Kozlowska M., Yu W., Baas P.W. (2010). Kinesin-12, a mitotic microtubule-associated motor protein, impacts axonal growth, navigation, and branching. J. Neurosci..

[17] Buster D.W., Baird D.H., Yu W., Solowska J.M., Chauvière M., Mazurek A., Kress M., Baas P.W. (2003). Expression of the mitotic kinesin Kif15 in postmitotic neurons: Implications for neuronal migration and development. J. Neurosci..

[18] Zmasek C.M., Eddy S.R. (2001). ATV: Display and manipulation of annotated phylogenetic trees. Bioinformatics.

[19] Katoh K., Rozewicki J., Yamada K.D. (2017). MAFFT online service: Multiple sequence alignment, interactive sequence choice and visualization. Brief. Bioinform..

[20] Livanos P., Müller S. (2019). Division plane establishment and cytokinesis. Annu. Rev. Plant Biol..

[21] Müller S. (2019). Plant cell division-defining and finding the sweet spot for cell plate insertion. Curr. Opin. Cell Biol..

[22] Guo L., Ho C.M., Kong Z., Lee Y.R., Qian Q., Liu B. (2008). Evaluating the microtubule cytoskeleton and its interacting proteins in monocots by mining the rice genome. Ann. Bot..

[23] Richardson D., Simmons M., Reddy A. (2006). Comprehensive comparative analysis of kinesins in photosynthetic Eukaryotes. BMC Genom..

[24] Tomei E.J., Wolniak S.M. (2016). Transcriptome analysis reveals a diverse family of kinesins essential for spermatogenesis in the fern *Marsilea*. Cytoskeleton.

[25] Yamada M., Goshima G. (2017). Mitotic spindle assembly in land plants: Molecules and mechanisms. Biology.

[26] Lee Y.R., Liu B. (2000). Identification of a phragmoplast-associated kinesin-related protein in higher plants. Curr. Biol..

[27] Pan R., Lee Y.R., Liu B. (2004). Localization of two homologous *Arabidopsis* kinesin-related proteins in the phragmoplast. Planta.

[28] Lee Y.R., Li Y., Liu B. (2007). Two *Arabidopsis* phragmoplast-associated kinesins play a critical role in cytokinesis during male gametogenesis. Plant Cell.

[29] Vanstraelen M., Inzé D., Geelen D. (2006). Mitosis-specific kinesins in *Arabidopsis*. Trends Plant Sci..

[30] Tian S., Wu J., Li F., Zou J., Liu Y., Zhou B., Bai Y., Sun M.X. (2016). NtKRP, a kinesin-12 protein, regulates embryo/seed size and seed germination via involving in cell cycle progression at the G2/M transition. Sci. Rep..

[31] Tian S., Wu J., Liu Y., Huang X., Li F., Wang Z., Sun M.X. (2017). Ribosomal protein NtRPL17 interacts with kinesin-12 family protein NtKRP and functions in the regulation of embryo/seed size and radicle growth. J. Exp. Bot..

[32] Smertenko A., Assaad F., Baluška F., Bezanilla M., Buschmann H., Drakakaki G., Hauser M.T., Janson M., Mineyuki Y., Moore I. (2017). Plant Cytokinesis: Terminology for structures and processes. Trends Cell Biol..

[33] Gaillard J., Neumann E., Van Damme D., Stoppin-Mellet V., Ebel C., Barbier E., Geelen D., Vantard M. (2008). Two microtubule-associated proteins of *Arabidopsis* MAP65s promote antiparallel microtubule bundling. Mol. Biol. Cell.

[34] Ho C.M., Lee Y.R., Kiyama L.D., Dinesh-Kumar S.P., Liu B. (2012). *Arabidopsis* microtubule-associated protein MAP65-3 cross-links antiparallel microtubules toward their plus ends in the phragmoplast via its distinct C-terminal microtubule binding domain. Plant Cell.

[35] Müller S., Smertenko A., Wagner V., Heinrich M., Hussey P.J., Hauser M.T. (2004). The plant microtubule-associated protein AtMAP65-3/PLE is essential for cytokinetic phragmoplast function. Curr. Biol..

[36] Ho C.M., Hotta T., Guo F., Roberson R.W., Lee Y.R., Liu B. (2011). Interaction of antiparallel microtubules in the phragmoplast is mediated by the microtubule-associated protein MAP65-3 in *Arabidopsis*. Plant Cell.

[37] Kosetsu K., de Keijzer J., Janson M.E., Goshima G. (2013). MICROTUBULE-ASSOCIATED PROTEIN65 is essential for maintenance of phragmoplast bipolarity and formation of the cell plate in *Physcomitrella patens*. Plant Cell.

[38] Hancock W.O. (2014). Mitotic kinesins: A reason to delve into Kinesin-12. Curr. Biol..

[39] Müller S., Jürgens G. (2016). Plant cytokinesis—No ring, no constriction but centrifugal construction of the partitioning membrane. Semin. Cell Dev. Biol..

[40] Jurgens G. (2005). Cytokinesis in higher plants. Annu. Rev. Plant Biol..

[41] Sasabe M., Machida Y. (2012). Regulation of organization and function of microtubules by the mitogen-activated protein kinase cascade during plant cytokinesis. Cytoskeleton.

[42] Ganguly A., DeMott L., Zhu C., McClosky D.D., Anderson C.T., Dixit R. (2018). Importin-β directly regulates the motor activity and turnover of a kinesin-4. Dev. Cell.

[43] Vavrdová T., Šamaj J., Komis G. (2019). Phosphorylation of plant microtubule-associated proteins during cell division. Front. Plant Sci..

[44] Petrovská B., Cenklová V., Pochylová Z., Kourová H., Doskočilová A., Plíhal O., Binarová L., Binarová P. (2012). Plant Aurora kinases play a role in maintenance of primary meristems and control of endoreduplication. New Phytol..

[45] Van Damme D., Bouget F.Y., Van Poucke K., Inzé D., Geelen D. (2004). Molecular dissection of plant cytokinesis and phragmoplast structure: A survey of GFP-tagged proteins. Plant J..

[46] Oh S.A., Allen T., Kim G.J., Sidorova A., Borg M., Park S.K., Twell D. (2012). *Arabidopsis* Fused kinase and the Kinesin-12 subfamily constitute a signalling module required for phragmoplast expansion. Plant J..

[47] Oh S.A., Johnson A., Smertenko A., Rahman D., Park S.K., Hussey P.J., Twell D. (2005). A divergent cellular role for the FUSED kinase family in the plant-specific cytokinetic phragmoplast. Curr. Biol..

[48] Oh S.A., Bourdon V., Dickinson H.G., Twell D., Park S.K. (2014). *Arabidopsis* Fused kinase TWO-IN-ONE dominantly inhibits male meiotic cytokinesis. Plant Reprod..

[49] Rasmussen C.G., Sun B., Smith L.G. (2011). Tangled localization at the cortical division site of plant cells occurs by several mechanisms. J. Cell Sci..

[50] Xu X.M., Zhao Q., Rodrigo-Peiris T., Brkljacic J., He C.S., Müller S., Meier I. (2008). RanGAP1 is a continuous marker of the *Arabidopsis* cell division plane. Proc. Natl. Acad. Sci. USA.

[51] Walker K.L., Müller S., Moss D., Ehrhardt D.W., Smith L.G. (2007). *Arabidopsis* TANGLED identifies the division plane throughout mitosis and cytokinesis. Curr. Biol..

[52] Schaefer E., Belcram K., Uyttewaal M., Duroc Y., Goussot M., Legland D., Laruelle E., de Tauzia-Moreau M.L., Pastuglia M., Bouchez D. (2017). The preprophase band of microtubules controls the robustness of division orientation in plants. Science.

[53] Stöckle D., Herrmann A., Lipka E., Lauster T., Gavidia R., Zimmermann S., Müller S. (2016). Putative RopGAPs impact division plane selection and interact with kinesin-12 POK1. Nat. Plants.

[54] Buschmann H., Dols J., Kopischke S., Peña E.J., Andrade-Navarro M.A., Heinlein M., Szymanski D.B., Zachgo S., Doonan J.H., Lloyd C.W. (2015). *Arabidopsis* KCBP interacts with AIR9 but stays in the cortical division zone throughout mitosis via its MyTH4-FERM domain. J. Cell Sci..

[55] Abu-Abied M., Belausov E., Hagay S., Peremyslov V., Dolja V., Sadot E. (2018). Myosin XI-K is involved in root organogenesis, polar auxin transport, and cell division. J. Exp. Bot..

[56] Arima K., Tamaoki D., Mineyuki Y., Yasuhara H., Nakai T., Shimmen T., Yoshihisa T., Sonobe S. (2018). Displacement of the mitotic apparatuses by centrifugation reveals cortical actin organization during cytokinesis in cultured tobacco BY-2 cells. J. Plant Res..

[57] Reichelt S., Knight A.E., Hodge T.P., Baluška F., Šamaj J., Volkmann D., Kendrick-Jones J. (1999). Characterization of the unconventional myosin VIII in plant cells and its localization at the post-cytokinetic cell wall. Plant J..

[58] Molchan T.M., Valster A.H., Hepler P.K. (2002). Actomyosin promotes cell plate alignment and late lateral expansion in *Tradescantia* stamen hair cells. Planta.

[59] Feng J., Hu Z., Chen H., Hua J., Wu R., Dong Z., Qiang L., Liu Y., Baas P.W., Liu M. (2016). Depletion of kinesin-12, a myosin-IIB interacting protein, promotes migration of cortical astrocytes. J. Cell Sci..

[60] Wu S.-Z., Bezanilla M. (2014). Myosin VIII associates with microtubule ends and together with actin plays a role in guiding plant cell division. eLife.

